# Enhancing research support services in health organizations by implementing a “Research Concierge Desk”, a case study

**DOI:** 10.3389/frma.2024.1335240

**Published:** 2024-04-05

**Authors:** Areej AlFattani, Abeer AlFirm, Norah AlBedah, Haifa AlDakhil, Elaf Al Muaythir, Leena Zeyad, Yasmin AlTwaijri

**Affiliations:** Department of Biostatistics, Epidemiology and Scientific Computing, King Faisal Specialist Hospital and Research Center, Riyadh, Saudi Arabia

**Keywords:** research concierge, data clinic, data management, services, collaboration, organization

## Abstract

Health organizations with teaching and research responsibilities face the need to establish a comprehensive system that addresses the processes and challenges associated with research activities; a system that assists local institutes in becoming research-active by identifying gaps and providing actionable recommendations. The involvement of epidemiologists, biostatisticians, and data scientists is paramount in offering technical and scientific support to health researchers. In our organization, research support services, such as technical, statistical, logistical, and scientific assistance, have been provided to researchers for the past 20 years under the name of “Data Clinic Service”. This article discusses the establishment of a physical booth called the “Research Concierge Desk” within a medical center to offer on-site, free-of-charge, and direct consultations to researchers, thereby improving accessibility to data clinic services. The underlying concept of the “Research Concierge Desk” is to align the research workflow for busy clinicians, who require vital assistance in the technical aspects of their research. As well, the desk and its digital platform enabled us to assess research process workflow, such as research submission, data clinic requests, research progress tracking, and researcher satisfaction assessment. We present the initiation of the “Research Concierge Desk” as an innovative solution in hospital settings, outline the available resources, benefits, challenges, and propose areas for improvement. The experience gained from implementing the “Research Concierge Desk” model can greatly benefit other health centers in adopting a similar approach to develop enhanced services for clinical researchers.

## Definitions

BESC: The Department of Biostatistics, Epidemiology and Scientific Computing.
Data Clinic: Name of the services that BESC is providing for clinical investigators.
Clients: The researcher who requires the service of the Data Clinic.
Service provider: The staff of BESC who is assigned to solve the required services.
Research concierge: The physical booth.
Data Clinic manager: The person from BESC who is responsible for assigning, managing, and tracking the requests of the data clinic.
RCA: Research CenterAdmin.

## Introduction

To ensure solid and right decisions, healthcare organizations that strive for excellence in services must use evidence-based methods and up-to-date research. Clinical research is one of the three pillars of excellence for any academic healthcare organization: healthcare, education, and research (Grol and Grimshaw, 1999). Successful research relies on the services' capacity to save the researcher time while providing a fluent and rich experience across the research lifecycle (Collura et al., 2019; Case Western Reserve University, 2023).

Biostatistics, data management, study design, and publication support are among the most demanded services because of their vital role in scientific advancements, driving translational research, and developing new methodologies for studying data collected through patient care and research. By improving infrastructure for the mechanism for conducting research and providing support to existing researchers, academic healthcare organizations can evolve into an excellent evidence-based level of patient care (Croghan et al., 2015). However, resources, individual and organizational capacity, and processes can function as either barriers or facilitators of research production (Collura et al., 2019). There is a pressing need to implement some changes in systems and processes to better assist clinical researchers in navigating data support networks (Friesen and Comino, 2017; Fynn et al., 2021).

In academia, research is a vital responsibility expected from academic staff, and librarians play a crucial role in facilitating academic research due to their expertise in data discovery, capture, description, analysis, curation, and visualization (Oelschlegel et al., 2018). Recognizing the significance of research support, many universities have focused on developing strategies to streamline the research workflow and provide comprehensive support services to their faculty and staff (Gutzman et al., 2018).

Kent State University in Ohio, for example, has an approach to developing a new research support system based on user feedback. To begin with research, Kent State University's academic library has provided the needed courses regarding reference management tools, data analysis, software support, acquisition services, publication, and personal services. Those services were offered to researchers through webpage information where the client can access the needed information through the institution's webpage (Larsen et al., 2010). The University of Central Florida Libraries has developed a service that integrates five departments at the institution to support research and data activities. These units include the library, research data management, the faculty center for teaching and learning, the office of research and communication, and the Institute for Simulation and Training. The model is a blend of a conceptual research lifecycle intertwined with specific service points. A webpage accesses these services by direct contact with assigned staff (Case Western Reserve University, 2023; University of Central Florida, 2023). Productivity metrics of library services in universities often focus on acquiring, cataloging, local dissemination, facilitating the production, publication of books and journals, and supporting authors (Larsen et al., 2010).

On the other hand, hospitals possess extensive patient data and registries, and they bear the responsibility of delivering optimal care based on evidence-based research (Sackett et al., 1996). However, clinicians and physicians often require comprehensive technical, epidemiological, ethical, and scientific support to effectively translate their research interests into publications (Arai et al., 2018). Availability and easy access to research services like that encourage them to initiate and conduct research besides their clinical duties. However, these services lack uniformity in processes and procedures in many health institutions, which makes it difficult to determine metrics of productivity (Rubio et al., 2011).

The experience of Duke's Office of Research at Duke University is valuable. The office developed a research management platform named “OnCore”. The platform is an integration of four systems, including Financials system—for budgeting and invoicing workflows for industry-funded protocols, the Duke's electronic health record, the electronic IRB system, and the general ledger. The overall goal was to create a standardized experience to manage clinical research from protocol creation to project closeout (Mullen et al., 2023). In this model, Duke's Office of Research was able to establish a solid system for research workflow that can be improved, maintained, and controlled over time.

In Saudi Arabia, King Faisal Specialist Hospital & Research Center (KFSH&RC) is a tertiary healthcare organization with centers in Riyadh, Jeddah, and Madinah. It is globally recognized for its world-class facility and excellent medical care provided by over 100 experienced doctors and 13,500 healthcare providers from different nationalities. KFSH&RC provides various professional development programs offered to internal and external employees, interns and students of different academic institutions.

As a leading global medical center, and aligning with 2030 Saudi Vision, one of its strategic objectives includes the aim “To be a knowledge leader through education, research, and innovation to support our goals and bring value to Saudi Arabia”. As such, the hospital management has mandated efforts toward encouraging physicians to include research output within their primary responsibilities. KFSH&RC focuses primarily on research in the fields of oncology, organ transplantation, cardiovascular diseases, neuroscience, and genetic diseases (KFSHRC, 2015).

The Department of Biostatistics, Epidemiology, and Scientific Computing (BESC) was established in 2000. It offers research-informed biostatistical, epidemiological, and data management support through a “Data Clinic Service”. These *pro bono* services have been instrumental over the years for a wide variety of KFSH&RC staff, including consultants, fellows, residents, interns, nursing, allied health, and administrative personnel. The purpose of these services is to enable researchers to focus on their scientific work by covering the technical aspects of research work (see Table 1). The Research Center Administration (RCA) launched a physical booth named the (*Research Concierge Desk*) which acted as an easy access service hub for researchers who needed guide or professional on-site consultation related to their research. In this article, we discuss our experience in facilitating the research workflow process for busy physicians by launching the “Research Concierge Desk” to provide more access to Data Clinic Services while also improving the services' workflow. We address the concierge desk's resources, the issues it faces, and suggest some methods to improve Data Clinic Services' operations. This experience could be adopted by medical institutions and hospitals to better support clinical research.

**Table 1:** Data clinic services offered by the BESC department.

**List of data clinic services**	
Research design	Data entry
Statistical analysis plan	Interviewing
Sample size calculation	Manuscript review
Proposal writing and review	Respond to reviewers' remarks support
Literature review	Data exporting and converting
Validation of questionnaire	Computational biology
Questionnaire design	Hardware specification
REDCap	Summary tables
Statistical analysis	Presentation graphics
Reference management (e.g., EndNote)	Software installation and maintenance
Randomization	Selecting appropriate journals
Regulatory registration SFDA	Educational courses throughout the year about many topics in clinical research
Proposal submissions	Facilitate collaboration among departments or institutions'
Chart abstraction	Provide research volunteers or trainees
Data management	Market for clinical trials and ongoing research projects

### Procedures and resources

The goals of the *Research Concierge Desk* are, first, to facilitate and expedite consultations with the required experts in clinical research without the need to contact multiple persons. Second, to provide sustainable value through tailored and customized services for a researcher based on their specific research needs. Third, to act as an information hub for the *Data Clinic Services* provided by the Biostatistics, Epidemiology, and Scientific Computing (BESC) Department. And forth, to link researchers to the Research Center's resources and to connect clinical investigators to interested collaborators and volunteers.

The Research Concierge Desk is located in the main hallway of the hospital, right in front of a large auditorium and beside the Research Center Administration (RCA) offices. The concierge was launched on the 1st of November 2019. The Chief Executive Officer, Chief Medical Officer, RCA leaders, and department chairs were invited to celebrate the ribbon-cutting ceremony. The place has high traffic and good visibility throughout the day. It is responsible to receive researchers, to answer research queries, and to guide them to apply for Data Clinic Services provided by BESC staff. The Department of BESC at KFSH&RC—the owner of this initiative- is staffed with professionals in essential research areas. Specialists like biostatisticians, epidemiologists, research data managers, research coordinators and registrars, research project managers, data scientists, and scientific programmers were willing to get collaboration requests from researchers.

In regards to manpower, at least 20 well-trained BESC staff members, epidemiology interns, were needed to cover the working hours of the concierge. To keep the concierge desk fully occupied, a schedule of names, times, and dates was generated, given that every staff member has a 2-h-long shifts per week. The covering staff were trained to respond to the frequently asked questions and give a brief promotional talk about the offered services. Many of the visiting employees said that it was the first time they had heard about the support that BESC offers to researchers. We asked visitors to sign their names with the minimum information in the visitor log for marketing purposes. Later on, we launched electronic announcements about the concierge and its services to be presented on screens across the whole organization, and then we sent generic emails to all medical staff all over the hospital regularly. As well, we made customized advertising by conducting several promotion presentations with the clinical departments during their staff or morning meetings.

In terms of logistics, the desk booth was custom-designed to fit the existing decorations around the area with a modern touch. Some furniture was necessary, including a table, three chairs, two iPad stands, a digital screen, and a large banner with a list of services. Additional equipment was brought like a computer, tablet, a telephone. Other miscellaneous items were brought for promotion purposes, like brochures, gift pens, flash USBs, and business cards.

To better use the technology, the leaders of the concierge desk initiative designed a digital platform through REDCap (Research Electronic Data Capture), to be used as an application submission channel for the required services. The researcher is required to provide his or her name, identification number, contact information, a brief about the research idea, and what is the required service. There is an option for attachments of related documents, if needed. This electronic form has been executed as a QR code and printed on business cards and on the wall of the physical concierge desk. As well, a webpage for the *Research Concierge Desk* and *Data Clinic Service* was designed and linked to the homepage of the hospital to explain the service and support access for users outside KFSH&RC.

To connect the client's request with the work of the BESC staff (the service provider), we designed a second online form. *The service provider form* is meant to be filled out by the staff of BESC who delivered the service to the researcher. It includes information about the type of connections they had with client (e-mail, telephone, face-to-face meetings, virtual meetings, or through them all). It includes an encoded turnaround time calculator to estimate, in hours or days, the time spent to deliver the service from assignment until closure of the request. Also, the service provider may explain completed tasks and insert an attachment to document the work.

Finally, an online “*researcher satisfaction survey”* was designed to be sent to the researcher once the provider completed and submitted *the service provider form*. Some information's gathered from this form were used as productivity metrics for BESC services.

**Figure 1:**
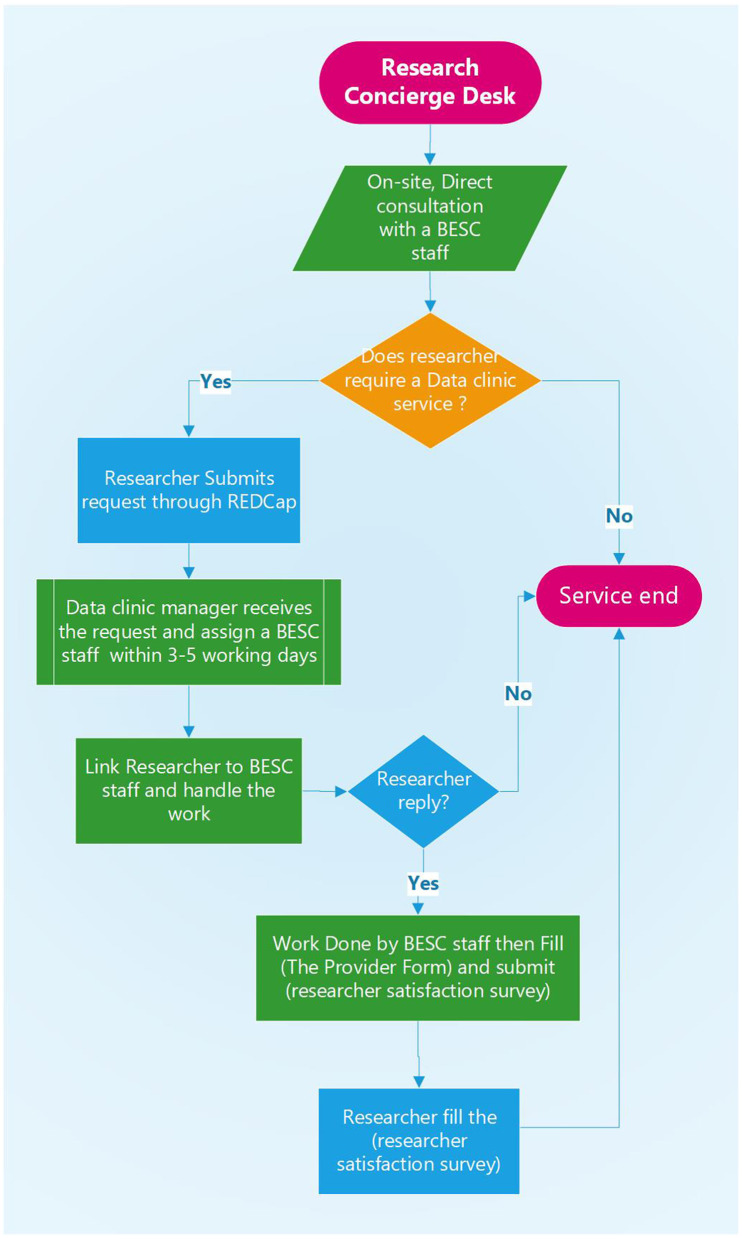
The role of research concierge and workflow of data clinic service.

The *researcher satisfaction survey* includes questions about how satisfied the client was about the service, whether the provider responded in a timely manner, and whether he/she was able to fully address the issue of the client. It includes a space for comments, complaints, or suggestions. Such indicators would be valuable to monitor the quality and quantity of the service, to track the requests, and to report it as productivity measures for BESC. The workflow of the data clinic services is shown in Figure 1, and a case study is presented in Box 1.

Box 1 **A case study for the role of research concierge desk and the data clinic services.**

A pharmacist came to the concierge desk to ask if a specific service was available. She wanted to convert data from a Case Report Form (CRF) related to a research study to be implemented in REDcap for having a more functional database. The covering BESC staff at the concierge discussed the issue with pharmacist, they agreed to proceed with a data clinic service. The BESC staff guided her to fill out the REDCap application and explained to her that she would receive a reply from the data clinic manager to connect her with the best-fit service provider.

Then the manager received the request, responded to it within two working days, and assigned a service provider from BESC who has the experience, ability, and time to help the pharmacist and then he sent an email to connect them together (the pharmacist and service provider).

The assigned service provider (i.e., BESC staff) met and discussed a work plan with the pharmacist, then they had a couple of follow-up meetings to review and assure accurate implementation of the CRFs in the REDCap platform.

Once done, the provider filled in the “Service Provider Form” to document ways of communication, meetings, and the time spent in this service. Hence, an automatic *researcher satisfaction survey* was sent to the pharmacist to collect her feedback. The pharmacist indicated the name of the service provider. She gave her opinion about general satisfaction and indicated the quality and timeliness of the work. Later on, that data was used in a clinical research article and published in a Q1 journal.

This model of service workflow could be used in other medical centers when a physical booth is not accessible. A telephone hotline to a medical center's research unit can serve as a research concierge; a staff member with extensive experience in research workflow can handle calls, act on them, or refer them to a more experienced one; and any online survey platform can function similarly to our REDCap application form. However, privacy and confidentiality concerns must be addressed. As a result, we advise other institutions that want to facilitate research for their personnel to use the model described here and customize it as needed.

Soon after the concierge desk opened, the COVID-19 pandemic began, and the demand for direct consultation and data clinic services rose owing to interest in COVID research. During the pandemic's lockdown periods, the concierge leader assigned electronic access through (Microsoft Teams) to a BESC staff member designated to cover the concierge, allowing researchers to continue receiving direct consultations. During the 2 years of the pandemic, this control action assisted many researchers in bringing the service totally online with high quality and prompt response.

### Data reports

We were able to recognize the most typical visitors based on departments, services, and job titles by extracting data from the Research Concierge desk's REDCap database. Statistical analysis (29%), and data management (11%), study design (10%), data collection (6.6%) are the most frequently requested services, followed by randomization, proposal preparation and review, SFDA registration, and software installation and maintenance.

Medical residents (17%), consultants (16.4%), and fellows (13.4%) made up the majority of researchers who requested data clinic service. Nurses, research coordinators, infection control coordinators, and interns made up the remaining researchers (33%). Pediatrics (12%), pharmaceutical care (8.6%), and medicine (7.3%) were the most popular departments that requested data clinic services. In terms of service purpose, 89% were for publication, 15% for presentation, and 20% for graduation project.

In terms of scientific activities, 581 research projects were accepted in the 2 years prior the concierge's desk (2018–2019), and this number climbed to 610 after 2 years (2020–2021). There were around 500 data clinic requests made through REDCap each year; 95% of them were allocated to a BESC expert within 3.5 working days, and 80% were completed and closed within 1–3 months.

We gathered fast feedback from the concierge's visitors: 80% of guests enjoyed the concept of the concierge desk, and 89% stated they were serviced efficiently.

## Discussion

The research concierge desk is a novel concept among medical institutions in the kingdom. We had improved visibility and access to our Data Clinic Service; we found some areas for improvement in data clinic workflow; and we were able to define some productivity measures for BESC's research services. We were able to deliver direct consultations from the research concierge totally online during the COVID-19 pandemic, indicating that our concept can be used by other medical institutions regardless of the existence of a physical concierge.

We faced several challenges. First, was client-related challenges, such as poor communication or insufficient documents to work on, or no response from clients after assigning data clinic tasks. Second, some research-related services are not offered in the data clinic due to a shortage of staff like scientific writers and manuscript reviewers. These services are in high demand among researchers who have had to outsource the editing phase. Third, there was limited awareness among medical staff about the service, although our marketing efforts, because the hospital had a high number of transferring or turnover medical staff all around the year, which makes it harder to reach everyone.

Similar challenges were identified in the Kent State University categories as institutional, personal, and technological. The challenges from an institutional perspective appear in the lack of communication about the sufficiency of the service itself, or they can be personal, as in finding the right staff and making sure the research concierge does not affect their professional role and position. Moreover, the challenges could be technological, as in the long period of adaption when applying a new technological solution (Collura et al., 2019).

### Areas of improvement

From our experience, we could identify some areas that we can work on improving the engagement of researchers in our data clinic services. First, internally, invest in empowering BESC service providers to be trained to deliver more services. For example, research coordinators can be trained to help in writing the manuscripts, which gives them the advantage of being eligible for authorship, plus they will help in providing one of the most needed services. Second, follow up with open client requests as we noticed clients are overwhelmed with the clinical work, which might take them away from tracking the research activities, so a reminder might push the cycle faster. Third, implement new marketing strategies, such as sending customized emails to active researchers, or research coordinators, or education program directors. Fourth, use the relevant events for promotion, such as in-house research days, which happen frequently and were hosted by several clinical departments. Fifth, catch unrecorded data clinic requests that come to BESC from different sources, like personal contact, meetings, or phone calls. These requests were not documented through the REDcap database, which makes it difficult to track the time BESC staff spent serving other departments.

Finally, initiate the collaboration with the medical library, in addition to assist the researchers by providing access to scientific articles and books, librarians can offer educational consultations and hands-on-training for literature review and reference management to be delivered on the concierge (Lewis et al., 1998).

### Recommendations

Implementing the idea of a research concierge service is a worthwhile pursuit for an institution of any size, and can be as versatile and robust as deemed necessary. The ultimate goal of the service is to eliminate or reduce potential delays, complications, or issues that researchers may encounter at any step of the research lifecycle (Lewis et al., 1998). An issue could take the form of a simple request that an individual is unsure who to address a query to, or it could also be a more perplexing, difficult issue that may require cross-unit coordination.

To expand the research portfolio, it is necessary to increase the competitiveness of the investigators for external funding. Research Development frequently engages with different corporate divisions to find new sources of finance for its research projects (Zier and Stagnaro-Green, 2001).

Some of the concierge services that have the potential to be included as:

- Searching for financing sources and providing advice on funding acquisitions (such as seminars and workshops) to assist researchers in developing winning grant proposals.
- Establishing connections between members and possible collaborators and mentors.
- Enhance collaboration with the medical library and a scientific writing center.

## Conclusion

In healthcare facilities, it is essential to formulate a strategy for the research support services that plan to achieve the institution's goals as well as fulfill researchers' needs. Our experience in “Data Clinic services” and the “Research Concierge Desk” can be taken as an example of dedicating resources for effective services, automating actions into interactive systems, marketing the goals (internally and externally), participating in networking and user activities. When developing new services, we recommend that special focus can be put on the quality of research data, which helps generate effective knowledge, yet encourages physicians to conduct scientific studies without fear of overwhelming duties.

## Author contributions

AAlFa: Writing – review & editing, Writing – original draft, Supervision, Methodology, Conceptualization. AAlFi: Writing – review & editing, Writing – original draft, Visualization, Methodology, Investigation. NA: Writing – review & editing, Writing – original draft, Visualization, Methodology, Investigation, Data curation, Conceptualization. HA: Writing – original draft, Visualization, Methodology, Investigation, Formal analysis, Conceptualization. EA: Writing – review & editing, Visualization, Methodology, Investigation, Formal analysis, Data curation. LZ: Writing – review & editing, Visualization, Software, Methodology, Investigation, Formal analysis. YA: Writing – review & editing, Writing – original draft, Supervision, Resources, Project administration, Methodology, Funding acquisition, Conceptualization.
